# Clinical characteristics and outcome of Tuberculosis lymphadenitis in a tertiary center from Saudi Arabia

**DOI:** 10.1016/j.jctube.2023.100384

**Published:** 2023-06-13

**Authors:** Ali Algarni, Nabih Alansari, Moayad Alqurashi, Mohammed Alsaeed

**Affiliations:** a Department of Medicine, Infectious Disease Division, Prince Sultan Military Medical City, Riyadh, Saudi Arabia; b Department of Medicine, Infectious Disease Division, King Fahad Armed Forces Hospital, Jeddah, Saudi Arabia; c Alfaisal University, College of Medicine, Riyadh, Saudi Arabia

**Keywords:** Tuberculosis, Tubercular lymphadenitis, Lymphadenopathy

## Abstract

**Introduction:**

Tuberculosis is among the deadliest infectious diseases. Lymphadenitis is an inflammation of the lymph nodes which is the most common extrapulmonary manifestation of tuberculosis. Saudi Arabia is rated as a country with a low incidence of tuberculosis. The study’s objective is to describe the clinical characteristics and outcome of TB lymphadenitis (TBL) at a large tertiary care center in Riyadh, Saudi Arabia.

**Method:**

All patients 18 years and older diagnosed with TB lymphadenitis between 2010 and 2021 at a single tertiary center in Riyadh, Saudi Arabia, were reviewed retrospectively for their clinical presentation, diagnostic yield, therapy, and outcome.

**Result:**

107 patients were included in the final analysis. The distribution of males and females were nearly equal, at 50.5 % and 49.5 %, respectively. The average age was 45. During the ten-year period of our investigation, the number of confirmed TBL ranged from 19 (the highest in 2010) to as little as one patient in 2021. 72.8 percent of patients presented with TBL affecting the neck. The most commonly used diagnostic method was histopathological examination of the tissue sample, granulomatous inflammations were found in 89.2 % of cases of theses necrotizing granuloma. 10.7 % of our isolates had resistance. The average duration of anti-TB treatment was 6.8 months with a cure rate of 72.9 %.

**Conclusion:**

The majority of patients in this study had cervical lymphadenopathy, with histopathology being the mainstay of diagnosis. 90 % of TBL cultures were susceptible to first-line anti-TB therapy.

## 1 Introduction

Tuberculosis (TB) is a common infection that affects 9.1 million people annually and kills 1.5 people each year despite being a curable disease, making TB the deadliest infectious disease [1]. Lymphadenitis is an inflammation of the lymph nodes, and it’s the most common presentation of TB outside the lung [2].

The clinical characteristics and outcomes of TBL have been discussed in numerous studies from around the world. The clinical presentation of lymphadenitis varies, but the most frequent manifestation is an enlargement of the cervical lymph nodes [3], [4]. Systemic symptoms (fever, weight loss, and night sweats) have been described with varying frequencies; 75 % of patients in an Indian study exhibited fever [6]. In the majority of studies [6], [7], [8], females were more frequently diagnosed with TB lymphadenitis; however, in two studies, males were diagnosed at a higher rate [3], [9]. HIV and diabetes are commonly seen in patients diagnosed with TBL, with rates approaching 35 % and 10 %, respectively [9]. The majority of studies have relied on histopathology characteristics as the primary diagnostic tool for TB lymphadenitis [4], [7], [10], while other methods (acid fast bacilli and polymerase chain reaction) have a lower yield [3]. Some patients who lacked microbiological evidence were labeled in one study using clinical probability [3]. There are various approaches to treatment and total duration; in one study [8], the median duration time was 8.6 months, while in another study [9], all patients were treated for at least nine months. In 84 % of cases, the outcome was deemed successful [3].

Saudi Arabia is one of the largest countries in the Middle East, with a population of 31 million people based on the latest population data [11]. It has a TB incidence rate of 8.1 (6.9–9.1) per 100,000 in 2020 and is classified as a low incidence country for TB [12]. Only one study from Saudi Arabia describing the clinical-diagnostic experience of 99 patients with tuberculous lymphadenitis between 1983 and 1998 was found in the literature search. The study showed a median age of 38 and a preponderance of women. 80 % were affected in the cervical region, while 36 % had systemic symptoms. 90 % of patients had lymphadenopathy lasting longer than one month. The diagnostic yield of fine-needle aspiration was 46 %, while the yield of excisional lymph node biopsy was 98 %. There was no significant difference in outcome between treatment administered for 6 and 9 months, and 6 % of patients experienced paradoxical lymph node enlargement [5]. Our aim in this study is to describe the clinical characteristics and outcome of TBL in a large tertiary center in Riyadh, Saudi Arabia. We believe that the findings of our study could very well contribute to a better understanding of the epidemiology of TBL, as well as the diagnostic tool and treatment duration that could contribute to a more favorable outcome.

## 2 Materials and methods

This is a retrospective chart review conducted at Prince sultan Military Medical City, a tertiary medical facility with over 800 beds in Riyadh, Saudi Arabia. The protocol of the study was approved by the hospitals institutional review board. All patients 18 years of age and older diagnosed with TBL between January 2010 and December 2021 were analyzed. Patients who were younger than 18, had incomplete health records, other organ involvement (additional tests have been conducted to rule out) or did not complete treatment were excluded.

Cases were identified using the hospital TB registry database, a database of histology reports from the Pathology Department, and a database of positive *Mycobacterium tuberculosis* cultures from the Microbiology Laboratory. A confirmed case of tuberculous lymphadenitis was defined by a positive culture for *M. tuberculosis*, a positive acid-fast bacillus (AFB) smear, a positive TB PCR. Presumptive tuberculosis lymphadenitis is based on epidemiological factors together with physical findings, radiographic findings and/or positive tuberculin skin test (TST) or interferon-gamma release assay (IGRA) or histopathological features (granuloma) with no clinical evidence of other infectious or non-infectious diseases.

Charts were reviewed and the following data were extracted: age, gender, symptoms, body site of lymphadenitis, duration of symptoms, sites of extranodal involvement, image findings, results of FNA and/or excisional lymph node biopsy, and details of antituberculous treatment.

Data collection was done on an electronic data sheet using Microsoft Excel for Mac V16.68 (2019, Microsoft Corp, Redmond, WA, USA). Basic statistical analysis (means, medians, standard deviations) was done also using Microsoft Excel data sheet.

## 3 Results

### 3.1 Epidemiology

During the study period, a total of 151 cases of TBL were identified, of which 44 were excluded, as shown in Supplementary Figure 1. And hence, the total number of cases considered is 107. The mean age is 45, and the distribution of males and females is nearly equal at 50.5 % and 49.5 %, respectively. Table 1 summarizes the epidemiological characteristics of this cohort. The majority of our sample is Saudi (95.3 %), and the most prevalent comorbidities are diabetes (24.2 %) and chronic kidney disease (14.9 %), non were HIV. The calculated mean Charlson comorbidity index was 2. There was a wide variance in the number of new cases of TBL across the study’s 10-year time frame, from a high of 19 in 2010 to a low of a single case in 2021 (Supplementary Figure 2).

**Table 1:** Demographic variables and risk factors.

Gender	Male (%)	54 (50.5 %)
	Female (%)	53 (49.5 %)
Mean Age	All (SD)	45 (±19)
	Male (SD)	47 (±20)
	Female (SD)	43 (±18)
Nationality	Saudi (%)	102 (95.3 %)
	Non-Saudi (%)	5 (4.6 %)
Co-morbidity	DM (%)	26 (24.2 %)
	CKD (%)	16(14.9 %)
	Solid organ transplant (%)	1 (0.9 %)
	Solid tumor (%)	8 (7.4 %)
	Hematological malignancy (%)	6 (5.6 %)
**Mean Chrlison comorbidity index** (SD)		2 (±3)

SD: Standard deviation, DM: Diabetes Miletus, CKD: Chronic Kidney Diseases.

### 3.2 Clinical presentation

The clinical manifestations of TBL varied among patients. Table 2 summarizes the clinical characteristics that were comparable between males and females. By a huge margin, lymph node enlargement was the most prevalent presenting symptom in patients of both sexes (57.0 %), followed by fever (31.7 %), significant weight loss (28.9 %), and night sweats (20.0 %). The male group had a higher proportion of constitutional symptoms overall. TBL was discovered incidentally in 12 asymptomatic patients through imaging performed for another reason. The mean duration of symptoms was 102 days in the male group and 134 days in the female group.

**Table 2:** Clinical features.

		Male (%)	Female (%)	Total (%)
Clinical feature	Lymph node enlargement	30 (55.5 %)	31 (58.4 %)	61 (57.0 %)
	Draining siun/discharge	0 (0 %)	3 (5.6 %)	3 (2.8 %)
	Local pain	2 (3.7 %)	1 (1.8 %)	3 (2.8 %)
	Chest pain	1 (1.8 %)	0 (0 %)	1 (0.9 %)
	Abdominal pain	1 (1.8 %)	0 (0 %)	1 (0.9 %)
	Cough	3 (5.5 %)	2 (3.7 %)	5 (4.6 %)
	Hoarseness	0 (0 %)	1 (1.8 %)	1 (0.9 %)
	Shortness of breath	2 (3.7 %)	2 (3.7 %)	4 (3.7 %)
	Abdominal swelling	1 (1.8 %)	0 (0 %)	1 (0.9 %)
	Fever	19 (35.1 %)	15 (28.3 %)	34 (31.7 %)
	Weight loss	17 (31.4 %)	14 (26.4 %)	31 (28.9 %)
	Night sweats	13 (24.0 %)	9 (16.9 %)	22 (20.5 %)
	Fatigue	5 (9.2 %)	1 (1.8 %)	6 (5.6 %)
	Asymptomatic	6 (11.1 %)	6 (11.3 %)	12 (11.2 %)
**Mean Duration of symptoms in days** ± SD (IQR)		102 ± 137 (60)	134 ± 202 (78)	117 ± 172 (83)

SD: standard deviation, IQR: Interquartile range.

### 3.3 Lymph node group

Table 3 describes the lymph node characteristics for our population. We used the lymph node region to simplify the descriptions of lymph node groups. TBL most commonly affected the neck (72.8 % of patients) and the chest (45.9 % of patients), with frequency being similar between the sexes. 15.8 % of patients experienced pain in the axillary or abdominal region. Inguinal was the area found in the fewest people (4 males and 0 females). However, 42.0 % of patients presented with disseminated lymphadenitis (affecting multiple lymph node groups) and 57.9 % with TBL affecting a single lymph node group. In terms of lymph node size, only 6.5 % of cases had lymph nodes smaller than 1 cm in size, while 51.4 % of cases had lymph nodes between 1 and 3 cm in size, and 34.5 % of cases had lymph nodes larger than 3 cm in size.

**Table 3:** Lymph nodes characteristics.

		Male (%)	Female (%)	Total (%)
Lymph node Region	Neck	36 (66.6 %)	42 (79.2 %)	78 (72.8 %)
	Mediastinal	27 (50.0 %)	21 (39.6 %)	48 (45.9 %)
	Axillary	9 (16.6 %)	8 (15.1 %)	17 (15.8 %)
	Abdominal	12 (22.2 %)	5 (9.4 %)	17 (15.8 %)
	inguinal	4 (7.4 %)	0 (0.0 %)	4 (3.7 %)
Number of lymph node group	one group	29 (53.7 %)	33 (62.2 %)	62 (57.9 %)
	Two groups	17 (31.4 %)	17 (32.0 %)	34 (33.3 %)
	Three groups	7 (12.9 %)	3 (5.6 %)	10 (9.3 %)
	Four groups	1 (1.8 %)	0 (0 %)	1 (0.9 %)
Disseminated lymphadenitis *	25 (46.2 %)	20 (37.7 %)	45 (42.0 %)	
Lymph node size	<1 cm	4 (7.4 %)	3 (5.6 %)	7 (6.5 %)
	1–3 CM	21 (38.8 %)	34 (64.1 %)	55 (51.4 %)
	>3 cm	26 (48.1 %)	11 (20.7 %)	37 (34.5 %)
	Not reported	1 (1.8 %)	3 (5.6 %)	4 (3.7 %)

*Disseminated lymphadenitis defined as two groups of lymph nodes or more.

### 3.4 Diagnostic modality

In our center, four methods of diagnosing TBL were used; the most common was histopathological examination of the tissue sample; granulomatous inflammations were found in 89.2 % of these cases. Necrotizing granulomas were more frequent than non-necrotizing granulomas (64.0 % versus 25.2 %, respectively). Reactive findings (no granuloma) were found in 3.9 % of cases. Positive tissue culture was found in 67.3 % of the samples tested; 5 isolates were resistant to INH, 2 isolates were resistant to Pyrazinamide, and 1 isolate was resistant to Ethambutol, Ciprofloxacin, and Streptomycin. Overall, resistance was found in 10.7 % of isolates. Fifty-three percent of patients had a positive result using the PCR test for the tissue sample. The acid-fast bacilli stain examination was the least helpful in diagnosing TBL, being positive in 7 out of 98 samples tested. The microbiological test with the highest sensitivity was the culture (67.3 %). And the least sensitive was the AFB stain, with only 7.1 % sensitivity. Six patients were diagnosed based on clinical suspicion, with no biopsies performed. (Summary Table 4).

**Table 4:** Diagnostic methods.

		Male (%)	Female (%)	Total (%)
**Histopathology**	Reactive (no granuloma)	3/51 (5.8 %)	1/50 (2.0 %)	4/101 (3.9 %)
	Granulomatous inflammation	48/51 (94.1 %)	47/50 (94.0 %)	95/101 (89.2 %)
	Necrotizing granuloma	34/51 (66.6 %)	34/50 (68.0 %)	68/101 (64.0 %)
	Non-necrotizing granuloma	14/51 (27.4 %)	13/50 (26.0 %)	27/101 (25.2 %)
**Positive tissue AFB stain**		4/51 (7.8 %)	3/47 (6.3 %)	7/98(7.1 %)
**Positive tissue culture**		37/49 (75.5 %)	25/43 (58.1 %)	62/92 (67.3 %)
**Resistance**		3/37(8.1 %)	4/25(16.0 %)	7/65 (10.7 %)
**Positive tissue PCR**		7/13 (53.8 %)	7/13 (53.8 %)	14/26 (53.8 %)

Sensitivity of tests *		**Male** (%)	**Female** (%)	**Total (%)**
**AFB stain in tissue samples**		7.8 %	6.4 %	7.1 %
**Culture from tissue samples**		75.5 %	58.1 %	67.4 %
**PCR from tissue samples**		53.8 %	53.8 %	53.8 %

*Sensitivity was calculated using the following calculation (true positive/(true positive + false negative)).

AFB: Acid-fast bacilli, PCR: Polymerase chain reaction.

### 3.5 Treatment and outcome

Most of the cases were treated for a period of 6 months. The average duration of INH and rifampicin treatment was 6.7 and 6.9 months, respectively. Pyrazinamide and ethambutol were found to have been used for a mean of 2.1 and 2.2 months, respectively. The average duration of second-line treatment only was 5.3 months, with ten patients receiving moxifloxacin and one patient receiving streptomycin. The treating physician (infectious diseases physicians) described clinical outcomes at the end of treatment; the cure rate was 72.9 % and was higher in the female group than in the male group, at 79.2 % and 66.6 %, respectively. Relapse was observed in 2.8 % of cases, all-cause mortality within 1 year was observed in 5.6 % of cases, and 18.7 % of cases were lost to follow-up. Radiological follow-up tests were performed on 38 patients at our center; regression was the most common post-treatment follow-up imaging result, occurring in 60.5 % of cases; complete resolution was reported in 31.5 % of patients; and worsening and recurrence were observed in only 5.2 % and 2.6 % of patients, respectively. (Summary Table 5).

**Table 5:** Treatment and outcome.

		Male (%)	Female (%)	Total (%)
**Mean Anti-TB treatment duration in months**	**First-line anti-TB**			
	INH	6.7	6.6	6.7
	RIF	7.0	6.8	6.9
	PZA	2.1	2.3	2.1
	Ethe	2.2	2.3	2.2
	**Second-line anti-TB** *	6.0	4.8	5.3
Clinical outcome **	Cure	36 (66.7 %)	42(79.2 %)	78(72.9 %)
	Relapse	1 (1.9 %)	2 (3.8 %)	3 (2.8 %)
	Death within one year	5 (9.3 %)	1 (1.9 %)	6 (5.6 %)
	Lost follow-up	12 (22.2 %)	8 (15.1 %)	20 (18.7 %)
Radiological outcome ***	Complete Resolution	4/15(26.6 %)	8/23 (34.7 %)	12/38(31.5 %)
	Worsening	2 (13.3 %)	0 (0 %)	2/38 (5.2 %)
	Persistence	0 (0 %)	1 (4.3 %)	1/38 (2.6 %)
	Regression	9 (6.0 %)	14 (60.8)	23/38 (60.5 %)

*10 patients received moxifloxacin and one patient received streptomycin.

**Clinical outcome was based on clinical judgement of the treating physicians regardless of radiological outcome.

***Radiological outcome was based on the size of lymph nodes regardless of clinical outcome.

TB: Tuberculosis, INH: Isoniazid, RIF: Rifampicin, PZA: Pyrazinamide, Ethe: Ethambutol.

## 4 Discussions:

There was inconsistency in how TBL was described epidemiologically in the literature. Females were more likely to be diagnosed with TBL than men in USA, France, UK, Taiwan, Zambia, and India, but males were more likely to be diagnosed in a data collected from Qatar and Denmark. Mean ages at presentation varied across studies, with a 42-year-old reported in one research from Taiwan and a 20-year-old reported in another from India [13], [16], [17], [19]. While there was a significant drop in cases between 2020 and 2021, we suspect that this was related to the COVID-19 pandemic and anticipate it to rise again (Supplementary figure 2).

While one US study found that 11.0 % of TBL were HIV positive [13], we did not find any HIV-positive individuals in our population. This could be to the low prevalence of HIV or TB involving other organs. Charlson comorbidity index (CCI) scores of zero were recorded as the most often detected in 81.9 % of patients in the Danish study [3]. However, we found that the mean CCI was 2, indicating that our cohort had a higher frequency of comorbidities.

In contrast to the studies conducted in Qatar and Malaysia, where night sweats were the most common presenting symptom, and Denmark and Malaysia, where constitutional symptoms were more common [3], [9], [19], we found that the majority of patients in our study had enlarged lymph nodes.

The neck lymph node group was the most common site of involvement in our study, occurring in 72.8 % of cases; this was followed by the mediastinal lymph node group. The results we obtained concur with those of previous studies. A study conducted in Tunisia found that the cervical lymph node group was involved in 83.4 % of patients. This was followed by the axillary and mediastinal lymph node groups [7]. Lymph nodes were found to be between 1 and 3 cm in size in 51.4 % of patients, with larger than 3-centimeter lymph nodes being found in 34.5 % of patients. Our results suggest that TBL is linked to more significant lymph node enlargement and should be evaluated in cases where lymph node is large with a risk factor for TB.

In our study, the sensitivity of the AFB smear test was lower than in most of other studies, reaching 37 % in one study [20]. A French study demonstrated a sensitivity of 8 % for the AFB smear, similar to our results [14]. Similar to Asimacopoulos et al. [17], we detected culture positivity in 67.3 % of patients; other research has shown tissue culture positivity to be as low as 18 % and 10 % [21]. PCR test positivity was similar to the 55 % reported by Singh et al. [21]; due to the previously described low sensitivity of other methods of diagnosis, 89.2 % of our cases were diagnosed based on clinical and histological findings of granulomatous inflammation. Variability seen may be attributable to differences in expertise and/or resource availability.

The mean duration of anti-TB treatment was 6.8 months, which is consistent with the Infectious Disease Society of America (IDSA) recommendation of a 6-month duration of treatment [22]. Our study demonstrated a cure rate of 72.9 % compared to a successful treatment in 84.3 % of patients in the Danish study [3]. This could be related to higher comorbidity rates in our population. Relapse occurred in 2.8 % (3 patients) as opposed to the 9 % mentioned by Polesky et al. [23] and one case by Smaoui et al. [7].

## 5 Conclusion

TBL is a difficult disease to diagnose and treat. There is considerable variation in the clinical presentation, and the diagnosis requires invasive procedure with low yield, necessitating a specialized setting and careful consideration of risk factors. In our cohort the number of newly diagnosed cases is decreasing. Ninety percent of TBL cultures were susceptible to first-line anti-TB treatment. Greater consideration needs to be given to diagnostic methods with emphasis on adherence to treatment and proper follow-up.

## Declaration of Competing Interest

The authors declare that they have no known competing financial interests or personal relationships that could have appeared to influence the work reported in this paper.
