# Supplementary figures and images for: Clinical characteristics and outcome of Tuberculosis lymphadenitis in a tertiary center from Saudi Arabia
Source: J Clin Tuberc Other Mycobact Dis. 2023 Jun 13;33:100384. doi: 10.1016/j.jctube.2023.100384 (PMC10727992; doi:10.1016/j.jctube.2023.100384)

Supplementary Figure 1


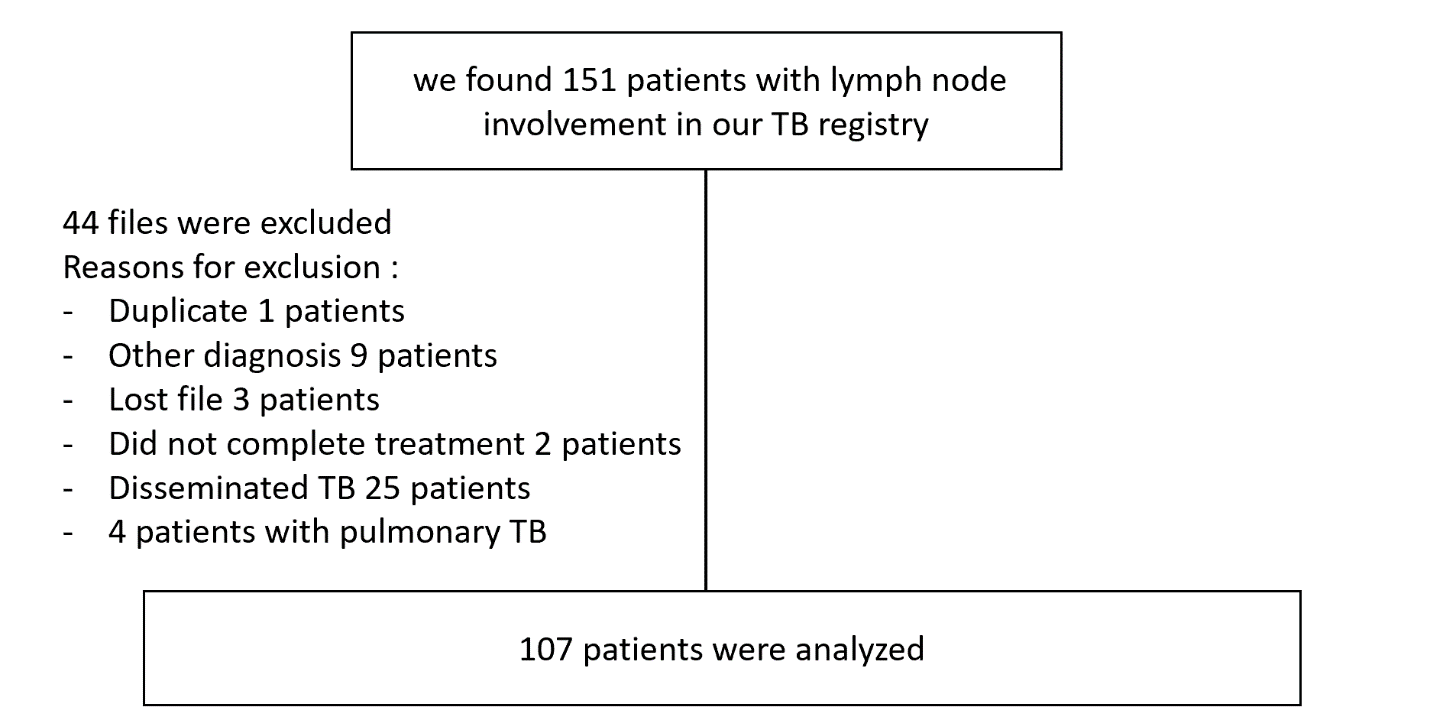


Supplementary Figure 2

Supplement: Supplementary data 1 [file mmc1.docx]
